# Global postural re-education in pediatric idiopathic scoliosis: a biomechanical modeling and analysis of curve reduction during active and assisted self-correction

**DOI:** 10.1186/s12891-018-2112-9

**Published:** 2018-06-21

**Authors:** Sarah Dupuis, Carole Fortin, Christiane Caouette, Isabelle Leclair, Carl-Éric Aubin

**Affiliations:** 1 Department of Mechanical Engineering, École Polytechnique de Montréal, P.O. Box 6097, Downtown Station, Station “Centre-ville”, Montreal, Quebec H3C 3A7 Canada; 2 Research Centre, Sainte-Justine University Hospital Centre, 3175 chemin de la Côte-Sainte-Catherine, Montreal, Quebec H3T 1C5 Canada; 3 School of rehabilitation, Faculty of Medicine, University of Montreal, 2900 Edouard-Montpetit, Montreal, Quebec H3T 1J4 Canada

**Keywords:** Scoliosis, Global postural re-education, Biomechanical modeling, Finite element model

## Abstract

**Background:**

Global postural re-education (GPR) is a physiotherapy treatment approach for pediatric idiopathic scoliosis (IS), where the physiotherapist qualitatively assesses scoliotic curvature reduction potential (with a manual correction) and patient’s ability to self-correct (self-correction). To the author’s knowledge, there are no studies regarding GPR applied to IS, hence there is a need to better understand the biomechanics of GPR curve reduction postures. The objective was to biomechanically and quantitatively evaluate those two re-education corrections using a computer model combined with experimental testing.

**Methods:**

Finite elements models of 16 patients with IS (10.5–15.4 years old, average Cobb angle of 33°) where built from surface scans and 3D radiographic reconstructions taken in normal standing and self-corrected postures. The forces applied with the therapist’s hands over the trunk during manual correction were recorded and used in the FEM to simulate this posture. Self-correction was simulated by moving the thoracic and lumbar apical vertebrae from their presenting position to their self-corrected position as seen on radiographs. A stiffness index was defined for each posture as the global force required to stay in the posture divided by the thoracic curve reduction (force/Cobb angle reduction).

**Results:**

The average force applied by the therapist during manual correction was 31 N and resulted in a simulated average reduction of 26% (*p* < 0.05), while kyphosis slightly increased and lordosis remained unchanged. The actual self-correction reduced the thoracic curve by an average of 33% (*p* < 0.05), while the lumbar curve remained unchanged. The thoracic kyphosis and lumbar lordosis were reduced on average by 6° and 5° (*p* < 0.05). Self-correction simulations correlated with actual self-correction (*r* = 0.9).

**Conclusions:**

This study allowed quantification of thoracic curve reducibility obtained by external forces applications as well as patient’s capacity to self-correct their posture, two corrections commonly used in the GPR approach.

## Background

Idiopathic scoliosis (IS) is a tridimensional (3D) spine deformity of unknown cause that alters body posture and affects perception of self-image and psychological confidence [1]. The deformation severity is conventionally evaluated with Cobb angle measurement on the coronal radiographs. Conservative treatments are recommended for small (10°–20°) and moderate deformities (20°–45°), which in North America traditionally consist of bracing and/or observation [1].

Since 2011, the international society on Scoliosis Orthopaedic and Rehabilitation Treatment (SOSORT) recognizes physiotherapy specific scoliosis exercises (PSSE) as a complementary or alternative conservative management to bracing [2]. More recently, the 2016 SOSORT guidelines have shown a higher strength of evidence regarding the efficacy of PSSE to prevent curve progression in IS [3]. Among them, Global Postural Re-education (GPR) aims to reduce postural impairments, regain back muscle symmetry and adequate posture through active muscular stretching postures, motor control and sensory integration exercises [4, 5]. The selection of the appropriate exercises is based on a 3-steps comprehensive evaluation focusing on 1) body morphology and symptomatology, 2) examination of muscle retractions associated with posture alterations [6] and 3) re-equilibration tests to assess back muscle flexibility and spine deformity correction (curve reducibility) [6, 7].

Like other PSSE, GPR treatment is personalized to the patient condition [8], and curve reducibility potential assessment orients the treatment planning, in a way similar to flexibility tests for surgical planning. Manual correction and self-correction are two re-equilibration tests that aims to achieve a passive and active momentary correction used in GPR for curve reducibility assessment [9]. Self-correction is also an integration exercise used at the end of a treatment session in order to progressively integrate changes in posture [9]. Manual correction involves the physiotherapist applying targeted force with his hands to the patient’s trunk to reduce scoliosis. This re-equilibration test aims to reach a posture that reduces momentarily the scoliotic deformation and to qualitatively assess trunk muscles stiffness as well as posture compensations. Self-correction is an active recruitment of trunk muscles by the patient in order to reduce the spine deformity, ideally in 3D. Self-correction also aims to assess the patient’s ability to integrate the correction, in real time and is an exercise common to many other PSSE programs [10].

Recent studies have shown a beneficial outcome from PSSE on scoliosis stabilisation and quality of life [11–13], although there is still a need for quantitative studies to confirm their effectiveness and further understand the biomechanical mechanisms [10, 14, 15]. There are very few studies regarding GPR applied to IS [6, 7, 11], hence there is also a need to quantify and better understand the biomechanics of GPR curve reduction postures.

Finite element models (FEM) are often used to biomechanically assess IS and brace treatments [16–21]. A personalized FEM was developed to simulate and optimize brace design for adolescent IS [22–25]. This model has the potential to be adapted for other conservative treatment simulations such as physiotherapist’s treatment.

The objective of this study was to biomechanically and quantitatively evaluate two re-education corrections (manual correction and self-correction) in pediatric idiopathic scoliosis using FEM combined with experimental testing.

## Methods

### Experimental study design

#### Patients

A total of 17 patients diagnosed with pediatric IS, aged 10.5 to 15.4 (one boy, 16 girls) and Risser sign 0 to 4, were recruited during their routine visit at our orthopedic clinic over a three months period. They all had a right thoracic major curve or a double major curve with right thoracic component, with a thoracic Cobb angle ranging from 11° to 45° (average 33° ± 9°), analytically measured between the perpendicular to the spine curve at its side change point near the end vertebrae. Their apical thoracic vertebral rotation ranged from 22° to − 6° (average 11° ± 9°), analytically measured using a 3D reconstruction method using pedicles and vertebral body orientation. One patient was excluded because the thoracic curve was less than 10°. Among the patients, seven were wearing thoraco-lombo-sacral orthosis (TLSO) and four were wearing nighttime braces. Two patients had done physical therapy in the past and six were still following PSSE or GPR treatment alone or in complement with their brace treatment. Patients filled a pain questionnaire (Numeric Pain Rating Scale NPRS-11) to ensure they had no physical contraindication to participate to the study. The study protocol was approved by the Institutional Ethics Committee of Sainte-Justine university hospital centre, and each patient and their parents signed a consent form.

#### Experimental protocol

**Fig. 1:**
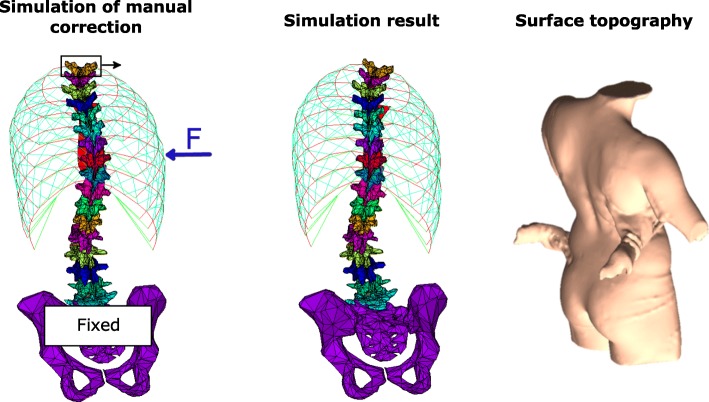
Manual correction simulation and measurement methodology

The recruited patients had their routine surface topography (InSpeck 3D Capturor, Creaform inc., Québec, Canada) and low dose biplanar radiographs (EOS™, EOS Imaging, Paris, France) taken in the presenting standing (reference) posture. A therapist who was trained specifically for this study (co-author IL) by a certified GPR physiotherapist (co-author CF) then performed the manual correction. To do so, the therapist stood behind the patient and applied a force with her right hand slightly under the right thoracic apex until a satisfying spine correction was obtained, while the left hand was positioned over the left iliac crest to stabilize the pelvis and overall posture (Fig. 1). The therapist wore gloves with force sensors (FlexiForce™ A301, sensitivity 5%, TekScan, Boston, MA, USA) to record the applied forces on the trunk surface. The sensors were sensitive to the normal force but not to shear forces. The posture was held for 10 s and repeated three times to obtain an average force value applied over time.

Then the self-correction posture was taught to the patient. When the posture was well understood and properly integrated, surface topography and biplanar radiographs were successively taken in this posture.

### Simulation methodology

The two GPR immediate corrections were simulated using a personalized FEM and compared to the clinical data collected for model verification purposes. For each patient, the personalized FEM was built in the reference standing posture and in the self-correction posture combining the surface topography and radiographs using Ansys 14.5 package (Ansys Inc., Canonsburg, PA, USA). The reconstruction method and FEM were initially developed and validated for brace wearing simulations [22, 23]. The model included the pelvis, sacrum, lumbar and thoracic vertebrae, ribs, costal cartilages, sternum, intervertebral disks and soft tissues. The mechanical properties were taken from previous published data and cadaveric studies [18, 26]. The model also included gravitational loads [27]. The model trunk rigidity can be personalized to the patient if an adequate radiograph is available (suspension, supine in traction, bending, etc.). However, ethical considerations precluded the obtention of these additional radiographs.

The manual correction was simulated by inputting the mean recorded force of the therapist’s right hand into the reference standing posture FEM. The pelvis was fixed in space to reproduce the therapist’s left hand action (Fig. 1). The first thoracic (T1) vertebra was allowed to move along the vertical axis, but was fixed in the sagittal plane at its reference standing position and was aligned with the Central Sacral Vertical Line (CSVL) in the coronal plane. These boundary conditions on T1 replace muscular forces generated by the patient to maintain his or her coronal alignment (righting reflex). The simulation allowed to calculate the curve reduction resulting from the external force exerted by the therapist.

**Fig. 2:**
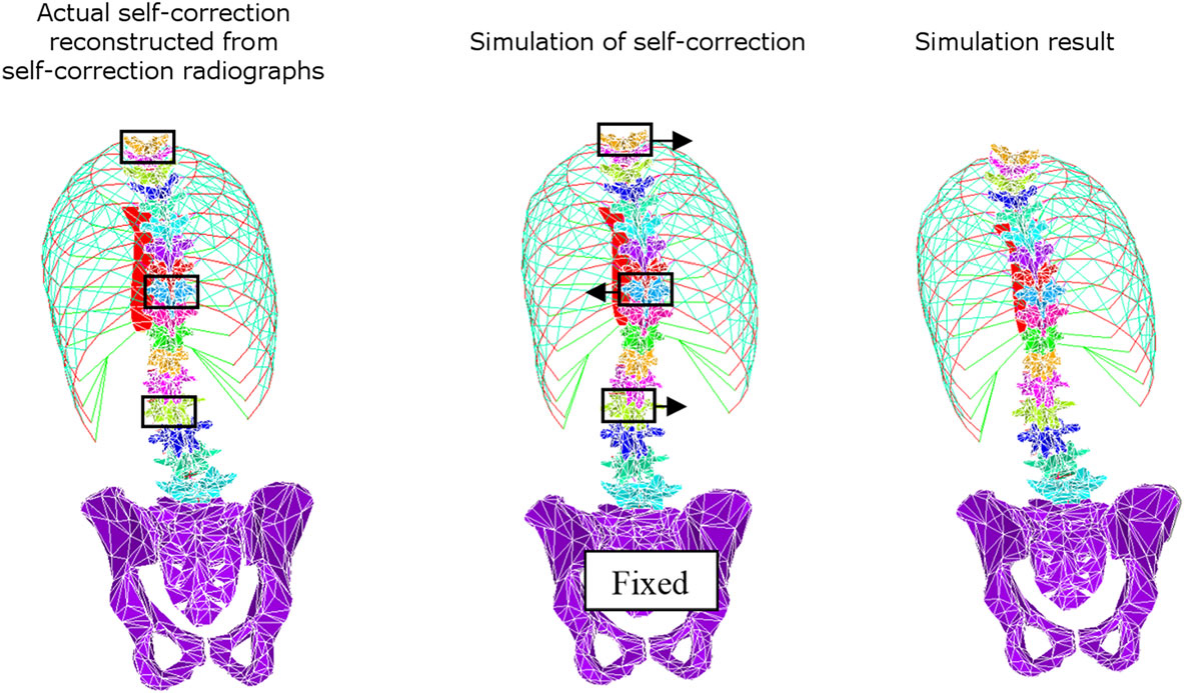
Self-correction was simulated by moving the position of T1 and of the thoracic and lumbar apical vertebrae of the reference FEM to their self-correction position as measured in the self-correction reconstruction from radiographs

To simulate the self-correction posture, the position of T1 and of the thoracic and lumbar apical vertebrae as measured in the sagittal and coronal radiographs were then applied to the reference FEM. The pelvis was fixed in space and the spine could move vertically (Fig. 2). The simulation allowed to calculate the thoracic curve correction and the reaction force at the thoracic apical vertebra required to achieve such correction. Self-correction simulation results were compared to the actual self-correction as documented with the acquired radiographs.

Indices were computed to quantify the patient’s ability to achieve scoliosis curve reduction during GPRs postures. For the manual correction, a stiffness index was defined as the ratio of the force applied at thoracic apex over the Cobb angle reduction (force/∆ Cobb angle). For the self-correction, the stiffness index was defined as the reaction force computed at thoracic apex over the thoracic curve reduction (force/∆ Cobb angle).

An ANOVA analysis with post-hoc Tukey test with 95% confidence interval was performed to compare the resulting Cobb angles using GraphPad Prism (GraphPad, La Jolla, CA, USA). Pearson coefficients were calculated to establish correlation between the different results. Differences and correlation with a *p* value < 0.05 were considered statistically significant.

## Results

Thoracic Cobb angles reduction and forces involved are summarized in Tables 1 and 2. The average equivalent force applied by the therapist during manual corrections over the thoracic curve was 31 ± 6 [23–55] N. The sensor under the main application point recorded an average pressure of 30.9 ± 14.1 [19.3–77.7] kPa. Manual correction simulations reduced the coronal thoracic curve on average by 26% [7–64%] (average Cobb angle correction of 8° [3°–19°], *p* < 0.05), while the coronal lumbar curve slightly increased compared to the reference standing posture (average of 27° [13°–42°] in reference standing posture vs. 29° [16°–43°] with the manual correction, *p* < 0.05). Thoracic kyphosis slightly increased while lumbar lordosis remained unchanged (kyphosis average of 30° [10°–47°] in the reference standing posture vs. 33° [11°–50°] with the manual correction (*p* < 0.05), lordosis average of 70° [57°–85°] in the reference standing posture vs. 71° [58°–85°] with the manual correction). The stiffness index ranged between 2 and 10 N/°. There was a significant correlation between the pressure applied by the therapist and the curve reduction obtained (*r* = 0.6, *p* < 0.05).

**Table 1:** Manual correction’s thoracic Cobb angle simulation results compared to reference standing posture and associated stiffness indices

Patient	Presenting deformity (standing posture)	Simulation of manual correction (% reduction)	Mean force applied by therapist over thoracic apex	Manual correction’s stiffness index
	deg	deg (%)	N	N / deg
P1	45	42 (7)	31	10
P2	25	21 (14)	33	10
P3	40	27 (31)	31	3
P4	33	25 (23)	28	4
P5	36	32 (13)	28	6
P6	23	16 (31)	23	3
P7	42	35 (17)	25	4
P8	36	27 (26)	25	3
P9	39	37 (7)	26	10
P10	28	10 (64)	55	3
P11	25	13 (48)	29	2
P12	37	31 (18)	33	5
P13	45	26 (43)	42	2
P14	11	5 (53)	31	5
P15	30	26 (13)	34	9
P16	31	27 (13)	28	7
Mean ± sd [min - max]	33 ± 9 [11–45]	25 ± 10 [5–42]	31 ± 8 [23–55]	5 ± 3 [2–10]

**Table 2:** Actual and simulated self-correction results of thoracic Cobb angle compared to reference standing posture and associated stiffness indices

Patient	Presenting deformity (standing posture)	Actual self-correction (% correction)	Self-correction simulation (% correction)	Reaction force at thoracic apex	Self-correction’s stiffness index
	deg	deg (%)	deg (%)	N	N/deg
P1	45	30 (33)	35 (22)	68	7
P2	25	19 (24)	21 (17)	27	6
P3	40	25 (37)	27 (33)	29	2
P4	33	23 (29)	24 (27)	22	2
P5	36	23 (35)	31 (15)	64	12
P6	23	16 (29)	12 (47)	8	1
P7	42	39 (7)	42 (2)	16	21
P8	36	17 (53)	27 (27)	52	5
P9	39	26 (34)	32 (19)	77	10
P10	28	22 (19)	25 (11)	24	8
P11	25	28 (−15)	28 (−13)	1	−0,40
P12	37	27 (29)	30 (19)	39	5
P13	45	29 (36)	30 (33)	79	5
P14	11	4 (61)	4 (66)	27	4
P15	30	9 (69)	15 (50)	100	7
P16	31	15 (53)	24 (25)	80	10
Mean ± sd [min - max]	33 ± 9 [11–45]	22 ± 9 [4–39]	25 ± 9 [4–42]	45 ± 30 [1–100]	7 ± 5 [0–21]

The actual self-correction reduced the thoracic curve deformity on average by 33% [from 69% reduction to 15% augmentation] (Cobb angle correction of 11° [− 4°–21°], *p* < 0.05), while the lumbar curve remained constant (average of 27° [13°–42°] in reference standing posture vs. 26° [14°–42°] with the self-correction posture). All patients except two reduced their thoracic curve by 19% or more; the two other patients had a smaller reduction of 3° (P7) or a slight increase of 4° (P12). The thoracic kyphosis and lumbar lordosis were reduced on average by 6° [from 17° reduction to 5° augmentation] and 5° [from 13° reduction to 6° augmentation] respectively (*p* < 0.05). Apical thoracic vertebral rotation ranged from 21° to − 13° (average 7° ± 11°). Self-correction simulations reduced the thoracic curve on average by 25% [from 66% reduction to 13% augmentation] (Cobb angle correction of 8° [− 3°–15°], *p* < 0.05)), while the lumbar curve remained constant. The thoracic kyphosis and lumbar lordosis were reduced on average by 7° [from 18° reduction to 5° augmentation] and 5° [from 12° reduction to 7° augmentation] respectively (*p* < 0.05)). There was a good correlation between the actual and simulated thoracic curve reduction with the self-correction (*r* = 0.9, *p* < 0.05). Simulated reaction force at thoracic apical vertebra was 45 N on average, resulting in a stiffness index between 0 and 21 N/°.

There was no correlation between the Cobb angle correction with the simulated self-correction and manual-correction (*r* = 0.1, *p* > 0.05).

## Discussion

This study allowed to quantify trunk stiffness in relation with thoracic curve reducibility as well as patient’s capacity to self-correct their posture through a clinical study and the use of a personalized FEM. Two different immediate correction mechanisms were analyzed to obtain a curve reduction depending on whether external force (manual correction) or active muscle recruitment solely (self-correction) are involved.

### Manual correction

To our knowledge this is the first study to report the forces applied by the physiotherapist over the trunk to manually correct the scoliotic deformities as part of a GPR approach. With no other existing adequate references, force and pressure ranges applied by the therapist’s right hand at thoracic apical vertebra were compared to reported values of force or pressure measured under thoracic pads of similar patients treated conservatively with orthopedic braces. Our results of force and pressure ranges are in agreement with studies on brace fitting [28–31]. Romano reported similar values of 25.9 [18.7–42.8] kPa with fiberglass braces in the sitting position with 17 patients [30], but Pham reported smaller pressure values with the Chêneau brace (average 8 kPa, 32 patients) [31], versus 30.9 kPa in the current study. Both Van den Hout ([4–209] N, 16 patients) and Périé ([0–113] N, 12 patients) studied Boston brace and reported a wide range of forces [28, 29], versus [23–55] N in the current study.

The stiffness index calculated allowed the development of a relative ranking according to the measured and simulated patient spine stiffness. We found a significant but moderate correlation between the force applied by the therapist and thoracic Cobb reduction (*r* = 0.6, *p* < 0.05) calculated in the FEM. Our findings are similar to a similar study of brace correction and measured pressures under brace pads (*r* = 0.9) (Wong (2000)) [32], but differ from the brace studies of Van den Hout (2002) (thoracic *r* = 0.5, lumbar *r* = 0.3) and Pham (2008) (*r* = − 0.08) [29, 31] that found smaller correlations. The cohort of Wong (2000) had flexible and correctable spines as documented by a supine lateral bending test, whereas this aspect was not specified in the two other studies. Because trunk rigidity is directly related to the slope of the correlation line, a moderate or low correlation could be explained by the variability in trunk rigidity between the cases that was not adjusted in the FEM. A two-factor correlation involving both the applied force and trunk stiffness related to thoracic correction would be interesting to calculate.

Manual correction simulation is coherent with the therapist’s empirical experience since it allowed to reduce momentarily the main curve deformity concurrently with a non-clinically significant 2° increase of the lumbar curve, under the recognized measuring error of 5° [33]. The lack of change of the lumbar curve was on purpose, because the main focus was to achieve the best correction possible of the thoracic segment with a pressure at the apical level without increasing the counter curvature. In GPR, this re-equilibration test aims to determine curve reducibility and the importance of body posture compensations to guide the clinician in the selection of active stretching postures and sensory integration exercises [9]. In the clinic, it is not possible to quantify the real correction. By having one hand on the thoracic region applying a corrective force while the other hand stabilizes the pelvis and overall posture, we can only limit upper trunk displacement by visual assessment. Radiographs could not be taken in this posture to verify the simulation results since the therapist stands behind the patient during this correction.

### Self-correction

**Fig. 3:**
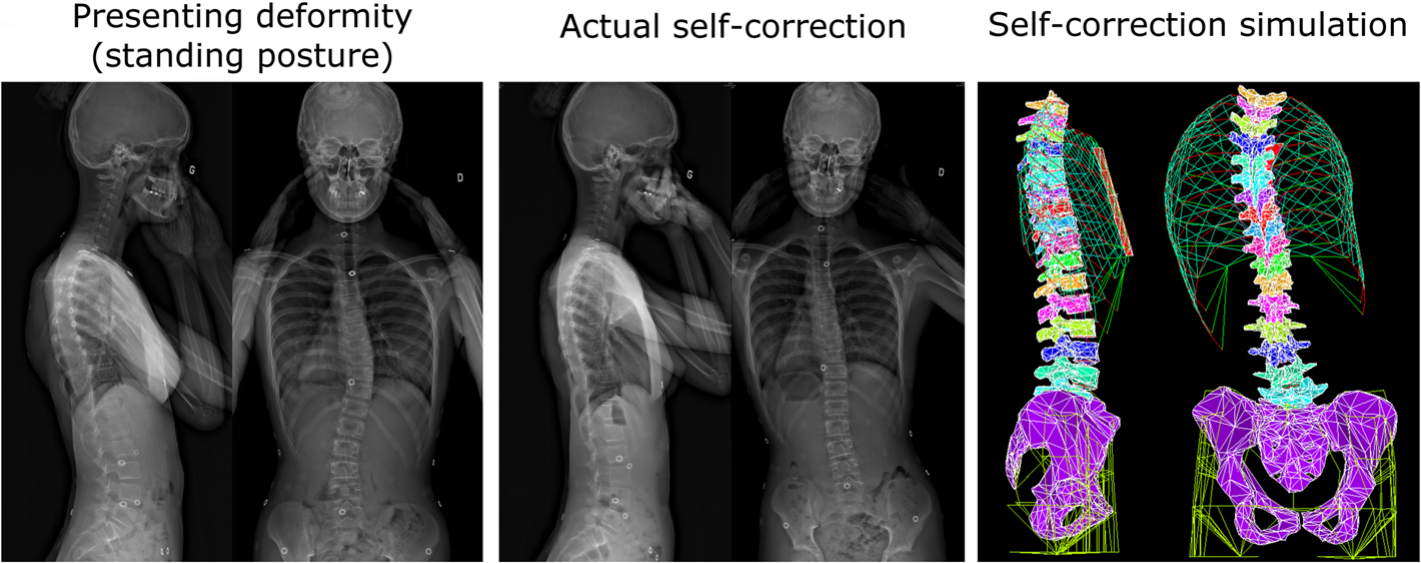
Low dose biplanar radiographs in standing posture and during self-correction illustrate patient’s potential to reduce the thoracic curvature immediately and momentarily. Self-correction numerical simulation agrees with self-correction radiographs

The self-correction resulted in a significant reduction of the main curve deformity indicating patient’s motor control ability for an immediate and momentary spine correction [11]. The main thoracic curve was corrected without accentuating the lumbar counter curve in the coronal plane, but a slight reduction of sagittal curves was measured. Different correction strategies were observed that lead to posture compensations, such as decreasing of the sagittal curves or accentuating the coronal slit as seen on Fig. 3. These observations suggest that self-correction exercises must be progressively integrated in the treatment and only when patients have a better body posture control to avoid negative side-effects on the long term. GPR treatment aims to progressively reduce the posture compensations while maintaining the achieved curve correction as the treatment progresses [9].

Self-correction tended to decrease vertebral rotation, going from 11° to 7° on average. Initial rotation was small for most participants, except for participants 12 and 13 who had a rotation of 22°. For these patients, the correction percentage in self-correction was still 29 and 36% respectively, indicating that high vertebral rotation does not preclude self-correction. The simulation of the self-correction underestimated the actual correction in the coronal plane, despite the good correlation (*r* = 0.9), which could be attributed to other muscular recruitment correction mechanisms not included in the simulation.

Reaction forces computed at thoracic apical vertebra could be associated to the muscle recruitment needed to maintain the correction and balance. Since the reaction forces are concentrated on three vertebrae (T1 and apical vertebrae) their values are higher than the actual distributed muscle recruitment forces along the spine to maintain the self-correction, which could explain the high force values obtained (Table 2). Still, using this reaction force, the simulation allowed to compute a stiffness index for a relative patient ranking according to their effort to maintain posture. Patient P7 had the lowest simulated correction (1°), therefore obtained the highest stiffness index (21 N/°), suggesting a high spine stiffness.

### Comparison between manual correction and self-correction

The absence of correlation between reductions obtained in the two postures suggests two different correction mechanisms. Manual correction is a passive correction, exclusively produced by the external forces from the therapist manipulation without active muscle recruitments. The obtained reduction hence is solely linked to the trunk inner resistance to the external force. On the contrary, self-correction is an active correction resulting exclusively from patient’s inner ability to autocorrect.

### Limitations

As for any computational model it is difficult to fully validate the FEM due to the limited data and standardized way to acquire it. For instance, for the manual correction, it was impossible to take radiographs while maintaining the posture. In the current study, simulations focussed on a specific subset of parameters such as the lateral force for the manual correction or apical vertebrae positions for the self-correction, which contributed to the complex mechanisms of correction exercises. While there are many unknowns in practice, numerical studies as the ones conducted in this study have the advantage of being able to evaluate the specific effects of the selected parameters. The various active muscle recruitment patterns, which may vary to assure a certain posture, were not tested. In this study, we only included the minimal forces needed to maintain a stable posture. Disk torsional rigidity was included in the FEM but not personalized to the patient-specific behavior, as it was not possible to measure using the available imaging data: this could affect the results for vertebrae with higher rotations, because intervertebral disc torsional rigidity increases with rotation [34]. Reproducibility of the inner forces needed for self-correction could not be assessed in this study due to the limited number of available radiographs, however self-correction reproducibility could eventually be measured by using a comparative non-irradiant method such as surface topography. Experimental limitations included different arm positions to comply with external topography and radiography protocols, which demanded additional attention from the patient to achieve self-correction and possibly altered their posture [35]. Hence even greater correction might be expected in self-correction during GPR treatment than during the current study.

### Clinical implications

The current study featured a small patient sample with a large range of curves; some patients were treated with braces and other were not. The results should therefore not be interpreted as a clinical evaluation of PSSE efficacy. Rather, this study highlights the feasibility of using FEM to better understand the effect of GPR correction postures. The computed stiffness index through the use of FE modeling allowed to quantify the passive (manual correction) and active (self-correction) resistance of the trunk and may contribute to set personalized therapeutic objectives for postural correction. The next step to standardize the stiffness index would be to have a constant manual pressure applied and observe the correction obtained.

## Conclusion

A FEM and experimental tools were developed to quantitatively assess two GPR curve reduction approaches and to better understand correction mechanisms. The forces exerted by the therapist during a manual correction approach, combined with the patient-specific FEM, allowed to simulate the scoliotic curve reduction. This would enable to quantify trunk stiffness without additional radiography. The self-correction simulation allowed to quantify the needed forces for the patient to reduce by him/her-self the main scoliotic spine curvature. Better understanding of correction mechanisms through GPR may help to support the contribution of this approach to scoliosis treatment.

## Abbreviations

3D: Tridimensional
CSVL: Central sacral vertical line
FEM: Finite element model
GPR: Global Postural Re-education
IS: idiopathic scoliosis
PSSE: physiotherapy specific scoliosis exercises
SOSORT: Scoliosis Orthopaedic and Rehabilitation Treatment
T1: first thoracic vertebra
TLSO: thoraco-lombo-sacral orthosis
